# The characteristics of lower extremity muscle activity and static balance of Chinese elite trampoline athletes during net contact phase

**DOI:** 10.3389/fphys.2025.1676910

**Published:** 2026-01-06

**Authors:** Kai Liu, Zhangbiao Zhu, Tong Yang, Jun Yin

**Affiliations:** 1 Department of Physical Education, Changzhi University, Changzhi, Shanxi, China; 2 Fujian Gymnastics Skills Management Center, Fuzhou, Fujian, China; 3 College of Sport and Physical Education, North University of China, Taiyuan, Shanxi, China; 4 Institute of Physical Education and Training, Capital University of Physical Education and Sports, Beijing, China

**Keywords:** trampoline, lower extremity muscle, static balance, plantar pressure, net contact phase

## Abstract

Lower extremity muscle strength and balance control are crucial components in high-level trampolining. However, current research on these aspects remains limited. This study aimed to explore the activity patterns of lower limb muscle groups and the balance characteristics of elite trampoline athletes in China, with a focus on comparing male and female athletes. Eleven elite trampolining athletes from the China national team (age: 23.42 ± 4.40 years; height: 165.37 ± 4.33 cm; weight: 53.38 ± 3.42 kg; training duration: 8.3 ± 2.61 years; M/F: 5/6) were required to test the electromyography (EMG), static balance, and plantar pressure. The Two-way ANOVA was adopted to compare the differences between sexes and lower limbs. The results showed significant gender differences in muscle activity during the net contact stage. Male athletes had higher Root Mean Square (RMS) values for the gastrocnemius (P < 0.01), with significant left-right differences in contribution rates (P < 0.05). In static balance, female athletes showed stronger right-side balance under closed-eye conditions (P < 0.05), while male athletes had stronger left-side balance under open-eye conditions (P < 0.01). Additionally, male athletes exhibited higher total plantar pressure on the left side (P < 0.01). This study reveals that during the net contact phase, male and female athletes exhibit distinct lower limb dynamics, with males showing a leftward shift in center of gravity and significant right ankle force, while both genders demonstrate heel-dominant plantar pressure and left-right balance differences, emphasizing the need for targeted unilateral balance and right ankle explosive strength training.

## 1 Introduction

Trampoline is a skill-intensive sport that emphasizes difficulty and aesthetics. Athletes must effectively use the trampoline’s resilience to perform complex movements (Aydın et al., 2023; Burt et al., 2016). Due to the unstable surface, precise control of body posture and balance is essential for executing these movements with accuracy. The net contact phase is crucial in trampolining, as it provides the necessary force and control for executing precise and high-difficulty aerial movements (Gómez-Landero et al., 2011; Takahashi et al., 2023; Huang et al., 2011). During pedaling, pre-activation of lower limb muscles is crucial for initiating aerial maneuvers, potentially leading to muscle imbalances with prolonged activation (Charbonneau et al., 2020). Studies have shown that male trampolinists frequently exhibit imbalances in the knee flexor-extensor muscle ratio, with similar imbalances observed in ankle joints during high-speed maneuvers (De Oliveira, 2021; Kellis and Katis, 2007; Siatras et al., 2004). Additionally, Chinese trampolinists demonstrate significantly low knee flexor-extensor muscle ratios, with stronger right lower limbs compared to the left (Li et al., 2012).

The functionality and balance of lower limb muscles are crucial for effective propulsion during the net contact phase. Asymmetry and imbalance caused by long-term specialized training are major contributors to sports injuries (Helme et al., 2021; Guan et al., 2022). Studies have shown that lower limb injuries are common among trampolinists, with an injury rate as high as 46.5%. Injuries to the ankle and knee account for 29.6% and 16.9% of cases, respectively (Zhu et al., 2014). Existing research on injuries in competitive trampolining primarily focuses on the injury location, injury rates in major competitions (Edouard et al., 2018), and demographic differences (Patel et al., 2021), with limited studies exploring the mechanisms of injury occurrence (Grapton et al., 2013). Although trampoline movements may appear symmetrical, significant muscle activation imbalances exist, which can hinder performance and increase the risk of injury (Rojas-Barrionuevo et al., 2017).

To prevent and reduce sports injuries caused by muscle imbalances, proper training and rehabilitation are essential. Studies show that functional strength training, balance exercises, and muscle stretching and relaxation can greatly improve lower limb muscle coordination and balance (Kordosi et al., 2014). In addition, real-time feedback systems based on sports biomechanics can monitor athletes’ posture and muscle activity, allowing for quick adjustments for movements and better muscle load distribution (Bahr and Krosshaug, 2005). Strengthening exercises for the knee and ankle joints, along with the use of protective taping, have also been shown to lower the risk of injury during high-intensity actions (Hr et al., 2022). These methods not only boost athletic performance but also help athletes extend their careers.

Since prior studies have explored trampoline techniques and injury rates, there is a critical lack of research on the detailed muscle mechanics and balance during the net contact phase. Therefore, this study aims to investigate lower limb muscular activity and static balance capacity during the net contact phase, providing scientific guidance and theoretical support for optimizing specialized training and injury prevention strategies. Based on previous studies, we hypothesized that significant lower limb asymmetries were observed between male and female athletes, and that these asymmetries differed between the sexes.

## 2 Methods

### 2.1 Participants

Eleven elite trampolinists from the Chinese national trampoline team in this study included. The cohort comprised five international-level competitors, six national-level competitors, and two individuals who achieved world championship titles (Table 1). The participants provided written informed consent for this experiment. All procedures and protocols were approved by the institutional ethical committee of the Capital University of Physical Education and Sports, Beijing, China (2019A05).

**TABLE 1:** Basic information of the participant.

	Height (cm)	Weight (kg)	Age (year)	Training duration (year)
Male (n = 5)	170.6 ± 2.61	58.26 ± 3.72	25.4 ± 4.56	9.2 ± 2.3
Female (n = 6)	160.14 ± 5.30	48.50 ± 3.10	21.43 ± 4.24	7.4 ± 2.9

### 2.2 Apparatus and measurement

**FIGURE 1:**
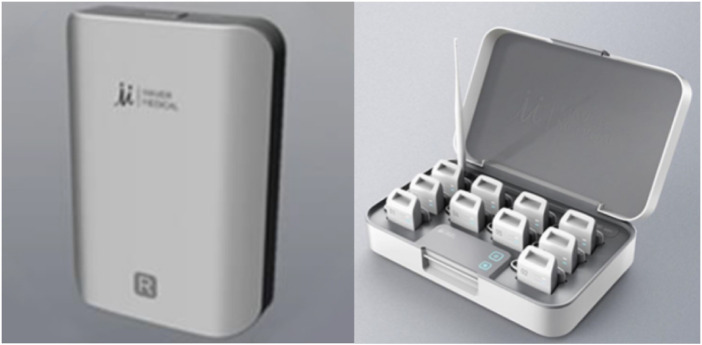
The LEOPARD and FREEDOM test system.

The electromyography (EMG) testing was conducted using the FREEDOM system from Shanghai Maiwo Medical Technology Co., Ltd. The sample frequency was set as 2000Hz. The static balance and plantar pressure assessments of trampolinists were conducted using the LEOPARD testing system (Shanghai Maiwo) (Figure 1).

Prior to testing, participants were briefed to ensure optimal performance during the assessments. The participants underwent hair removal and alcohol disinfection (75% ethanol) on relevant muscle regions. The tested muscles include rectus femoris, tibialis anterior, gastrocnemius, biceps femoris, and vastus lateralis bilaterally. Each participant was required to perform 10 vertical jump trials, during which pertinent EMG data were recorded. The execution of each movement was supervised to minimize measurement variability.

**FIGURE 2:**
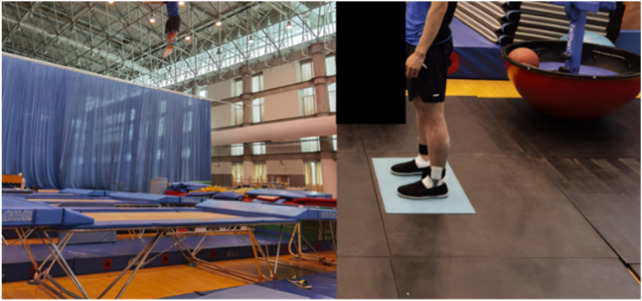
The Experiment environment (Left: lower extremity muscle activity test; Right: Static balance test).

After that, participants selected appropriate insoles based on their foot dimensions. The insoles operated at a frequency of 150 Hz. Both feet were fully positioned on the pressure platform. The whole test included six specific postures: both feet with eyes open, both feet with eyes closed, left foot with eyes open, left foot with eyes closed, right foot with eyes open, and right foot with eyes closed. Each posture was maintained for 30 s. All investigations were carried out under constant conditions (temperature controlled at 27 °C and kept quiet during the tests) (Figure 2). To assess the reliability of the measurement methods, repeated tests were conducted on a subset of participants (n = 5) across two separate sessions. The reliability of the EMG data was evaluated using the intraclass correlation coefficient (ICC), with results indicating excellent reliability (ICC = 0.85–0.95) across the recorded muscle activation levels. For plantar pressure measurements, the ICC ranged from 0.88 to 0.92, demonstrating high consistency in pressure distribution data. The static balance test showed an ICC range of 0.90–0.94, confirming the reliability of balance assessments across repeated trials.

### 2.3 Data analysis

All electromyography (EMG) signals were filtered with a Butterworth band-pass filter with a range of 20–500 Hz (Lu et al., 2024). All relevant information from the original EMG signals was retained. This study analyzed the Root mean square (rMS) and muscle contribution rate (MCR) of rectus femoris, tibialis anterior, gastrocnemius, biceps femoris, and vastus lateralis bilaterally.

$$RMS=\sqrt{\frac{\sum_{i=0}^{N}Data{\left[i\right]}^{2}}{N}}$$

$$MCR\left(\%\right)=\left(\frac{EMG\;activity\;of\;specific\;muscle}{Sum\;of\;EMG\;activity\;of\;all\;relevant\;muscles}\right)\times 100$$

**FIGURE 3:**
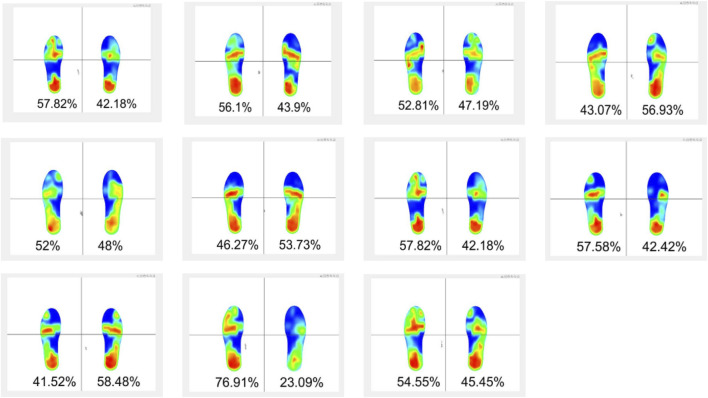
The Schematic diagram of plantar pressure test.

The distribution of plantar pressure was measured across various foot regions, including the left foot, right foot, former left ball, left mid-foot, left heel, former right foot, right mid-foot, and right heel. The following formulas were employed to calculate the pressure ratios: Left Foot (Left) Pressure Ratio = Total Foot (Left) Pressure/(Total Foot (Left) Pressure + Total Foot (Right) Pressure). Former Foot (Left) Pressure Ratio = Former Foot (Left) Pressure/(Former Foot (Left) Pressure + Mid-Foot (Left) Pressure + Heel (Left) Pressure) (Figure 3).

### 2.4 Statistical analysis

Statistical analyses were performed using Statistical Package for the Social Sciences (SPSS) (SPSS Statistics v 24, IBM Corp., US). For comparison of values, data distribution was analyzed for normality. Two-way ANOVA was used to compare the bilateral lower limb RMS values, muscle contribution rates, balance metrics, and plantar pressure values in sex and lower limbs. Statistical significance was assumed when *P* < 0.05. When significant differences were observed, Cohen’s effect size (ES) measures were computed.

## 3 Results

### 3.1 Results of RMS value

**FIGURE 4:**
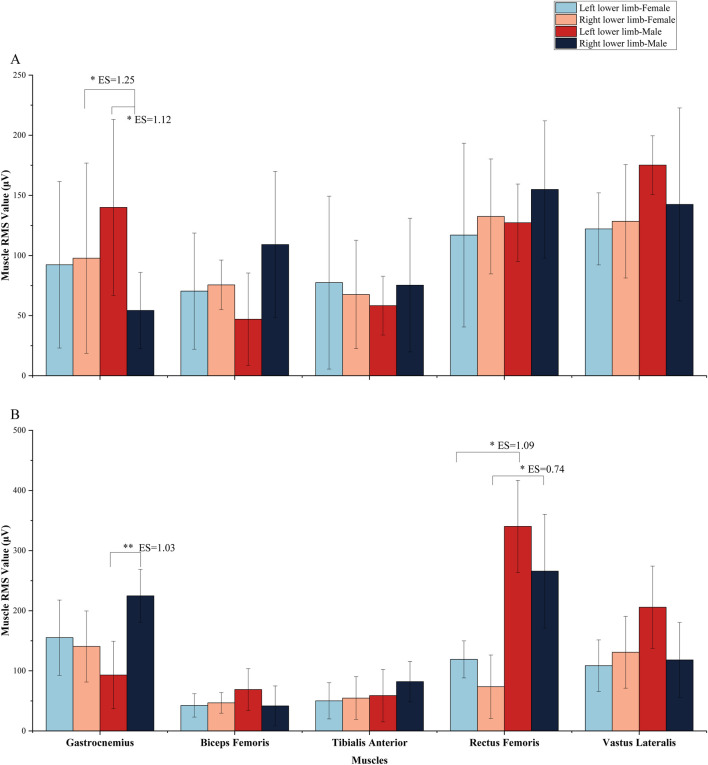
The results of muscle RMS value: **(A)** represents the result during the net touch phase; **(B)** represents the result during take-off phase.

During the net touch phase, the total RMS value of the right lower limb was higher than that of the left in male athletes (Figure 4A). Muscle activation levels (RMS values) in the right lower limb were greater than those in the left for all muscles except the gastrocnemius and vastus lateralis in male athletes. A significant difference in RMS values between the left and right gastrocnemius muscles was observed in male athletes (P < 0.05, ES = 1.12). Additionally, the RMS values of the gastrocnemius in the female right lower limb were significantly higher than those in male athletes (P < 0.05, ES = 1.25).

Furthermore, the total RMS value of the left lower limb was greater than that of the right in female athletes (Figure 4A). During the take-off phase, the total RMS value of the left lower limb was significantly greater than that of the right in female athletes (Figure 4B). The gastrocnemius showed a highly significant difference in female athletes (P < 0.01, ES = 1.03). The RMS value of the rectus femoris in the female left/right lower limb was significantly lower than that in males (P < 0.05, ES = 1.09; P < 0.05, ES = 0.74). Additionally, the total RMS value of the left lower limb exceeded that of the right in female athletes during the take-off phase (Figure 4B).

### 3.2 The results of muscle contribution rates

**FIGURE 5:**
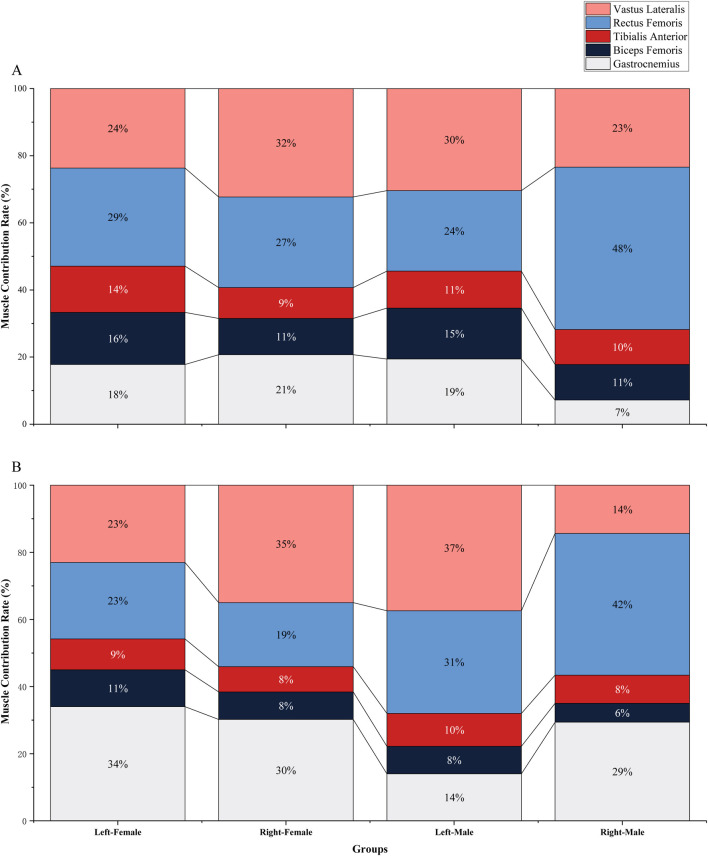
The results of muscle contribution rate: **(A)** represents the result during the net touch phase; **(B)** represents the result during take-off phase.

During the net touch phase, regarding the muscle contribution rates in female athletes, the left lower limb presented the following order: gastrocnemius, vastus lateralis, rectus femoris, biceps femoris, and tibialis anterior. For the right lower limb, the contribution rates were ranked as follows: vastus lateralis, gastrocnemius, rectus femoris, biceps femoris, and tibialis anterior. A comparative analysis of muscle contribution rates between the left and right sides demonstrated that only the vastus lateralis of the right lower limb had a higher contribution rate. In male athletes, the left lower limb exhibited the following order of muscle contribution: vastus lateralis, rectus femoris, gastrocnemius, tibialis anterior, and biceps femoris. For the right lower limb, the contribution rates were ranked as rectus femoris, gastrocnemius, vastus lateralis, tibialis anterior, and biceps femoris. The gastrocnemius showed a significant difference between the left and right lower limbs (P < 0.05, ES = 1.11). A comparative analysis revealed that only the gastrocnemius and rectus femoris in the right lower limb had higher contribution rates, while the remaining muscles exhibited lower contribution rates (Figure 5A).

During the take-off phase, in female athletes, the muscle contribution rates for the left lower limb were ordered as follows: rectus femoris, vastus lateralis, gastrocnemius, biceps femoris, and tibialis anterior. In contrast, the right lower limb showed the following order: vastus lateralis, rectus femoris, gastrocnemius, biceps femoris, and tibialis anterior. Compared to the left lower limb, the right lower limb demonstrated higher contribution rates of the gastrocnemius and vastus lateralis, with lower contribution rates of the remaining muscles (Figure 5B). For male athletes, the left lower limb displayed contributions in the following order: vastus lateralis, rectus femoris, gastrocnemius, biceps femoris, and tibialis anterior. Conversely, the contribution rates of the right lower limb were ranked as rectus femoris, vastus lateralis, biceps femoris, tibialis anterior, and gastrocnemius. Compared to the left lower limb, the right lower limb had higher contribution rates, excluding the rectus femoris (Figure 5B).

### 3.3 The results of balance test and plantar pressure

**FIGURE 6:**
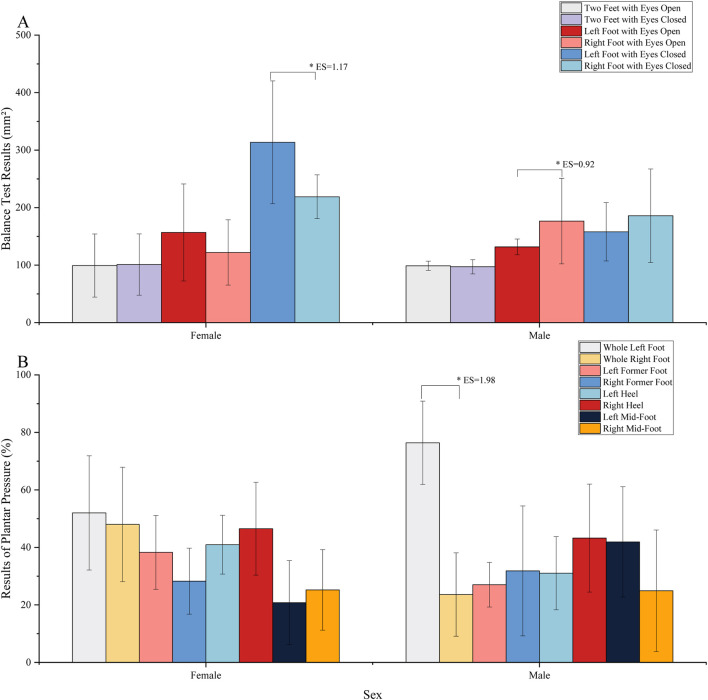
The results of balance test and plantar pressure: **(A)** represents the results of balance test; **(B)** represents the results of plantar pressure.

Female athletes demonstrate superior balance in the right lower limb compared to the left, with a statistically significant difference observed in the eyes-closed condition (*P* < 0.05, ES = 1.17). Conversely, male athletes exhibit stronger balance in the left lower limb relative to the right, with a highly significant difference noted in the eyes-open condition (*P* < 0.01, ES = 0.92) (Figure 6 A). The plantar pressure analysis reveals that male athletes have significantly greater plantar pressure in the left foot compared to the right, with a highly significant difference (*P* < 0.01, ES = 1.98). In terms of pressure distribution, female athletes exhibit the highest-pressure ratio for both heels. In contrast, male athletes present the highest mid-foot pressure ratio in the left foot and the highest heel pressure ratio in the right foot (Figure 6 B).

## 4 Discussion

This study revealed that during the net touch phase, the rectus femoris, vastus lateralis, and gastrocnemius exhibited the highest RMS values, highlighting the quadriceps' dominant role in force generation. Significant lower limb asymmetries were observed, with male athletes showing greater left gastrocnemius activation and females demonstrating more balanced bilateral muscle contributions, which supported our hypnotizes. These imbalances suggest specific muscle groups play a critical role in stability and force production (Jensen et al., 2013), emphasizing the need for targeted conditioning to address asymmetries and improve performance while reducing injury risk (Dello Iacono et al., 2016; Schoenfeld, 2002).

These results indicate that the quadriceps serve as the primary muscle group for exertion during pedaling. Increased strength in the extensors of knee joints significantly contributes to maintaining the stability of the center of gravity (Rudolph, 2004; Park et al., 2021). The gastrocnemius plays a dual role: not only does it contribute to force generation, but its eccentric contraction also counteracts the trampoline’s rebound force (Hoang et al., 2007; Arampatzis et al., 2007). This interplay between the gastrocnemius and tibialis anterior is crucial for maintaining balance, despite the tibialis interiors’ secondary role in force production (Arampatzis et al., 2007). These observations suggest that while current training regimens focus heavily on primary exertion muscle groups, such as the quadriceps and gastrocnemius, antagonist muscle groups like the tibialis anterior are equally vital for sustaining balance and preventing injuries. Biomechanically, the gastrocnemius acts as both a propulsive muscle during the push-off phase and a stabilizer during the landing phase, while the tibialis anterior assists by controlling dorsiflexion and providing stability to the ankle joint. The anterior tibialis helps prevent excessive plantarflexion, which could otherwise lead to instability during landing. Moreover, the strength of these muscle groups is not only important for force production but also for minimizing injury risks. Weakness or imbalance between the gastrocnemius and tibialis anterior can lead to compensatory movements, which may increase the risk of strains or overuse injuries52.

Our results also revealed significant bilateral asymmetries in muscle activation and balance. Male athletes exhibited higher activation of the left gastrocnemius, with a pronounced leftward shift in their center of gravity. This imbalance was further corroborated by static balance and plantar pressure assessments, which indicated higher pressure and stability on the left side. Conversely, female athletes demonstrated more symmetrical muscle activation and balance between their lower limbs, although the right side exhibited slightly higher RMS values. These gender specific differences in muscle dynamics may reflect distinct biomechanical strategies for stabilizing the body during trampoline movements (Holden et al., 2016; Hu et al., 2023; McLean et al., 2007). Current training regimens for athletes predominantly emphasize primary exertion muscle groups, such as the quadriceps and gastrocnemius, often neglecting the antagonist muscle groups responsible for balance (Brachman, 2017; Zech et al., 2010; Hrysomallis, 2011). It is advisable for trampolinists to incorporate strength exercises for the anterior tibialis while focusing on balance training for the ankle flexor and extensor muscle groups.

The observed asymmetries raise concerns about injury risk, particularly for male athletes, where the imbalance between the left and right lower limbs may predispose them to overuse injuries. Previous research has shown that an asymmetry exceeding 15% significantly elevates injury risk (Grindem et al., 2012). Therefore, tailored conditioning programs should aim to mitigate these disparities by incorporating unilateral strength and balance training.

Furthermore, the static balance and plantar pressure assessments showed a predominant concentration of pressure in the heels, suggesting a strategy to maximize elastic potential energy storage in the trampoline. This heel-dominant approach increases dorsiflexion at the ankle, allowing greater deformation of the trampoline surface and more effective energy transfe (Yi-lin, 2015; Ya-Wei and Jing-Guang, 2011). The higher plantar pressure on the left side in male athletes aligns with their elevated RMS values, indicating the left lower limb’s primary role in stabilizing the body during the net touch phase. In contrast, female athletes demonstrated superior right-side balance with eyes closed, emphasizing the stabilizing function of the right lower limb.

Gender differences in lower limb stability among trampoline athletes highlight unique biomechanical and neuromuscular characteristics (Flaxman et al., 2014; Decker et al., 2003). Male athletes tend to rely more on their left leg for stabilization during dynamic movements, as shown by higher left-side plantar pressure and muscle activation (Hughes and Dally, 2015), while female athletes display better right-side balance in static tasks, likely due to higher sensitivity in joint position and a lower center of gravity (Fain et al., 2021; Hansen et al., 2019). These differences affect muscle use, with women engaging more opposing muscles for joint stability and men focusing on force generation. To improve performance and prevent injuries, male athletes should work on balancing strength between both legs, while female athletes should focus on exercises that build dynamic balance and lower limb strength (Hansen et al., 2019). Tailored training programs can help address these needs and improve overall performance. To improve lower limb asymmetries and enhance ankle stability, it is recommended to incorporate unilateral training and ankle strength exercises. Unilateral training, such as single-leg balance, single-leg squats, and single-leg deadlifts, helps improve lower limb strength and stability, especially when performed on unstable surfaces (Baudry et al., 2014). Additionally, lateral lunges and crossover step-ups can strengthen lateral movements, reducing the risk of injury. Ankle strength exercises, including calf raises, eccentric heel drops, and ankle circles, help reinforce the ankle muscles, improving stability and control. Combining these training methods will enhance performance and reduce the risk of injury in athletes (Yin et al., 2021).

Some possible limitations should be acknowledged. Firstly, although this study discusses injury risks related to muscle imbalances, it does not include actual injury data or follow-up to confirm the link between the identified asymmetries and injury incidence. Due to the lack of injury data, the results can only be inferred from a physiological and biomechanical perspective, and cannot directly establish a causal relationship between muscle imbalances and injury risk. Secondly, the reliance on static balance and plantar pressure assessments may not fully capture the dynamic balance challenges unique to trampolining. In trampolining, balance control involves not only static stability but also rapid shifts in center of gravity and coordination, which static tests may fail to fully represent. Additionally, the study’s small sample size and its limited control conditions in a laboratory setting may affect the generalizability of the results. Future research should aim to expand the sample size, incorporate dynamic assessments, and include real-world data to gain a more comprehensive understanding of the relationship between muscle imbalances and injury risk.

## 5 Conclusion

During the net touch phase, trampolinists exhibit peak quadriceps activation, with females demonstrating balanced lower limb exertion and males showing a leftward shift in center of gravity, where the left ankle plays a crucial role in stabilizing the body. In the take-off phase, the gastrocnemius is key, particularly for males, who generate significant force at the right ankle, highlighting the need for explosive strength training. Plantar pressure analysis reveals heel dominance and significant left-right asymmetries in both muscle activation and balance, underscoring the importance of unilateral strength and balance exercises to optimize performance and minimize injury risk.

## Data Availability

The raw data supporting the conclusions of this article will be made available by the authors, without undue reservation.
